# Reliability of pulse pressure and stroke volume variation in assessing fluid responsiveness in the operating room: a metanalysis and a metaregression

**DOI:** 10.1186/s13054-023-04706-0

**Published:** 2023-11-08

**Authors:** Antonio Messina, Mariagiovanna Caporale, Lorenzo Calabrò, Giulia Lionetti, Daniele Bono, Guia Margherita Matronola, Andrea Brunati, Luciano Frassanito, Emanuela Morenghi, Massimo Antonelli, Michelle S. Chew, Maurizio Cecconi

**Affiliations:** 1 Department of Anaesthesia and Intensive Care Medicine, IRCCS Humanitas Research Hospital, Via Manzoni 56, 20089 Rozzano - Milan, Italy; 2 Department of Biomedical Sciences, Humanitas University, Via Rita Levi Montalcini 4, 20072 Pieve Emanuele, Milan, Italy; 3 Department of Anesthesia and Intensive Care, Fondazione Policlinico Universitario ‘A. Gemelli’ IRCCS, Università Cattolica del Sacro Cuore, Rome, Italy; 4 Department of Anaesthesia and Intensive Care, Biomedical and Clinical Sciences, Linköping University, Linköping, Sweden

**Keywords:** Pulse pressure variation, Stroke volume variation, Fluid responsiveness, Fluid therapy, Hemodynamic monitoring

## Abstract

**Background:**

Pulse pressure and stroke volume variation (PPV and SVV) have been widely used in surgical patients as predictors of fluid challenge (FC) response. Several factors may affect the reliability of these indices in predicting fluid responsiveness, such as the position of the patient, the use of laparoscopy and the opening of the abdomen or the chest, combined FC characteristics, the tidal volume (Vt) and the type of anesthesia.

**Methods:**

Systematic review and metanalysis of PPV and SVV use in surgical adult patients. The QUADAS-2 scale was used to assess the risk of bias of included studies. We adopted a metanalysis pooling of aggregate data from 5 subgroups of studies with random effects models using the common-effect inverse variance model. The area under the curve (AUC) of pooled receiving operating characteristics (ROC) curves was reported. A metaregression was performed using FC type, volume, and rate as independent variables.

**Results:**

We selected 59 studies enrolling 2,947 patients, with a median of fluid responders of 55% (46–63). The pooled AUC for the PPV was 0.77 (0.73–0.80), with a mean threshold of 10.8 (10.6–11.0). The pooled AUC for the SVV was 0.76 (0.72–0.80), with a mean threshold of 12.1 (11.6–12.7); 19 studies (32.2%) reported the grey zone of PPV or SVV, with a median of 56% (40–62) and 57% (46–83) of patients included, respectively. In the different subgroups, the AUC and the best thresholds ranged from 0.69 and 0.81 and from 6.9 to 11.5% for the PPV, and from 0.73 to 0.79 and 9.9 to 10.8% for the SVV. A high Vt and the choice of colloids positively impacted on PPV performance, especially among patients with closed chest and abdomen, or in prone position.

**Conclusion:**

The overall performance of PPV and SVV in operating room in predicting fluid responsiveness is moderate, ranging close to an AUC of 0.80 only some subgroups of surgical patients. The grey zone of these dynamic indices is wide and should be carefully considered during the assessment of fluid responsiveness. A high Vt and the choice of colloids for the FC are factors potentially influencing PPV reliability.

**Trial Registration:** PROSPERO (CRD42022379120), December 2022. https://www.crd.york.ac.uk/prospero/display_record.php?RecordID=379120

**Supplementary Information:**

The online version contains supplementary material available at 10.1186/s13054-023-04706-0.

## Introduction

Fluid administration in the operating room is a cornerstone of perioperative hemodynamic optimization [1–4], and its titration is obtained by adopting the fluid challenge (FC) to assess preload dependency and avoid fluid overload. In patients under mechanical ventilation, dynamic indices such as stroke volume variation (SVV) and pulse pressure variation (PPV) reliably predict the effect of FC because the fixed and repetitive inspiratory and expiratory pressure changes affect right ventricle’s preload, afterload and, hence, stroke volume (SV).

In the last decade, different aspects of the use of PPV and SVV in the operating room have been further investigated, providing clinically relevant implications. First of all, PPV and SVV reliability is affected by specific validity criteria including a tidal volume (Vt) > 8 ml/kg, a normal right ventricle’s function, the absence of heart arrythmias, an heart rate/respiratory rate ratio > 3.6 and an unimpaired respiratory mechanics [5–7], becoming clinically useful only below or above a grey zone of uncertainty [8]. The majority of these criteria are usually respected in the operating room, with the exception of a protective Vt, which seems to be associated with better outcomes [9] and is now suggested as standard ventilation in the operating room [10]. This is clinically meaningful, since reducing the average Vt adopted in the operating room, also the threshold adopted in the past to stratify fluid responders and non-responders (i.e. 13% [11]) may be changed.

On the contrary, in the operating room other factors associated to the type of surgery may impact a lot on PPV and SVV reliability, such as the type of anesthesia adopted, the position of the patient, the use of laparoscopy (LPS) and the opening of the abdomen or the chest.

As second, the impact of different determinants of the FC itself (i.e. the volume, the rate, the type of fluid used and the threshold to define fluid responsiveness [12–14]), have been further investigated and, as consequence, the value of PPV and SVV in studies adopting different types of FC may be inconsistent.

We, therefore, conducted a comprehensive systematic review and metanalysis with the primary aim of investigating the performance of PPV and SVV in different surgical setting, stratifying the patients according to chest/abdomen opening, intraoperative position, and the use of LPS.

Secondarily, we assessed the impact of other potential factors influencing the reliability of these indices in predicting fluid responsiveness in mechanically ventilated patients in the different surgical settings, specifically FC characteristics, the modality of ventilation and the type of anestestia.

## Material and methods

We adhered to the *Preferred Reporting Items for Systematic Reviews and Meta-Analysis – Protocols* (PRISMA-P) guidelines [15] (Additional file 1: Table S1). The protocol of this study was prospectively registered with the *International Prospective Register of Systematic Reviews* (PROSPERO) (CRD42022379120).

### Data sources and search strategy

A systematic literature search was performed including PUBMED® and EMBASE® and the Cochrane Controlled Clinical trials register databases, by using the following terms: 'pulse pressure variation' OR 'stroke volume variation' OR 'fluid responsiveness' AND (surgery) OR (surgical patients) (Additional file 1: Table S2).

Articles written in English, enrolling at least 10 adult, mechanically ventilated patients undergoing elective surgery and published from 1st January 2000 until 1st March 2023 in indexed scientific journals were considered. Editorials, commentaries, letters to editor, opinion articles, reviews, and meeting abstracts were excluded. References of selected papers, review articles, commentaries, and editorials on this topic were also reviewed to identify other studies of interest missed during the primary search. When multiple publications of the same research group/center described potentially overlapping cohorts, the most recent publications were selected.

We included only those studies clearly stating the threshold for defining fluid responsiveness as SV (or its surrogates) increase above a predefined limit. Articles including data collected in the postoperative period were excluded, while data recorded in the post-operative ICU just after the end of the surgery were included. Finally, we excluded studies performed during liver transplantation, in pediatric population and during pregnancy/labor.

### Data abstraction

Three couples of examiners independently performed the evaluation of titles and abstracts. The articles were then subdivided into three subgroups: “included” and “excluded” (if the two examiners agreed with the selection) or “uncertain” (in case of disagreement). In the case of “uncertain” classification, discrepancies were resolved by further examination performed by two expert authors (A.M. and M.Ce.). We used a standardized electronic spreadsheet (Microsoft Excel, V 14.4.1; Microsoft, Redmond, WA) to extract data from all included studies, recording: trial characteristics (*i.e.* number of centers, country), patient population (*i.e.* demographics, type of surgery, baseline illness severity scores), intraoperative monitoring and interventions (*i.e.* mechanical ventilation characteristics, monitoring technology used, FC characteristics).

### Risk of bias assessment in the included studies

#### Assessment of risk of bias in the included studies

The QUADAS-2 scale was used to assess the risk of bias of the included studies [16]. Two expert authors (A.M. and M.Ce.) independently examined the studies using predefined criteria, which are reported in the Additional file 1: Table S4:

For each criterion, the risk of bias was judged as high (3 points), unclear (2 points) or low (1 point). If the answers to all signaling questions for a domain were “yes,” then risk of bias was judged as “low”. If any signaling question was answered “no,” the potential risk of bias was defined as indicted in Additional file 1: Table S4. The sum of these points was used to calculate the global risk of bias. Studies were included in the highest risk of bias group if the sum of the points obtained by the risk of bias and applicability judgment assessment, was higher than the median value for all the studies [17].

### Statistical analysis

Descriptive analysis was carried out: the statistical unit of observation for all the selected variables was the single study and not the patient. Quantitative variables were summarized with means (standard deviations, SD) or medians (inter-quartile ranges, IQR) according to their distribution.

Patients were stratified in five main groups, according to the surgical characteristics at inclusion: (1) Patients enrolled with closed abdomen and chest; (2) Patients enrolled with closed abdomen and open chest (including sternotomy and thoracotomy); (3) Patients enrolled with open abdomen and closed chest; (4) LPS; (5) Prone position.

We adopted a metanalysis pooling of aggregate data with random effects models using the common-effect inverse variance model. The area under the curve (AUC) of pooled receiving operating characteristics (ROC) curves was reported with 95% confidence intervals (95%CI). In-between study heterogeneity was assessed with the I^2^ statistic. According to Higgins et al., I^2^ values around 25%, 50%, and 75% represented no, low, moderate, and high heterogeneity [18]. Unless stated otherwise, we considered the number of the FC performed equal to the number of patients included in the study. In the studies comparing two different surgical settings in the same population (i.e., open chest/closed chest, supine/prone etc.) data of the two subgroups of patients were separately analyzed for the purpose of the ROC curve analysis. Missing data in AUC reporting was considered an exclusion criterion from metanalysis.

For each of these five subgroups we performed a meta-regression considering the following independent variables: 1) Tidal volume (Vt) ≥ 8 ml/kg; 2) Positive end-expiratory pressure (PEEP) level (*i.e.,* PEEP = 0 cmH_2_O; PEEP = 0–5 cmH_2_O; PEEP > 5 cmH_2_O); 3) total intravenous anesthesia (TIVA); 4) FC using colloids vs crystalloids; 5) volume of FC administration > 4 ml/kg; 6) rate of FC administration > 15 min).

In case of mixed populations (i.e. receving TIVA anesthesia/alogenates or undergoing LPS /laparotomy), the subgroup including at least 75% of the population was used for the final classification of the study.

The statistical analysis was performed using the software STATA® version 17 (StataCorp, College Station, TX, USA) and Medcalc (Software 8.1.1.0; Mariakerke, Belgium). For all comparisons, we considered significant *p* values < 0.05.

## Results

**Fig. 1:**
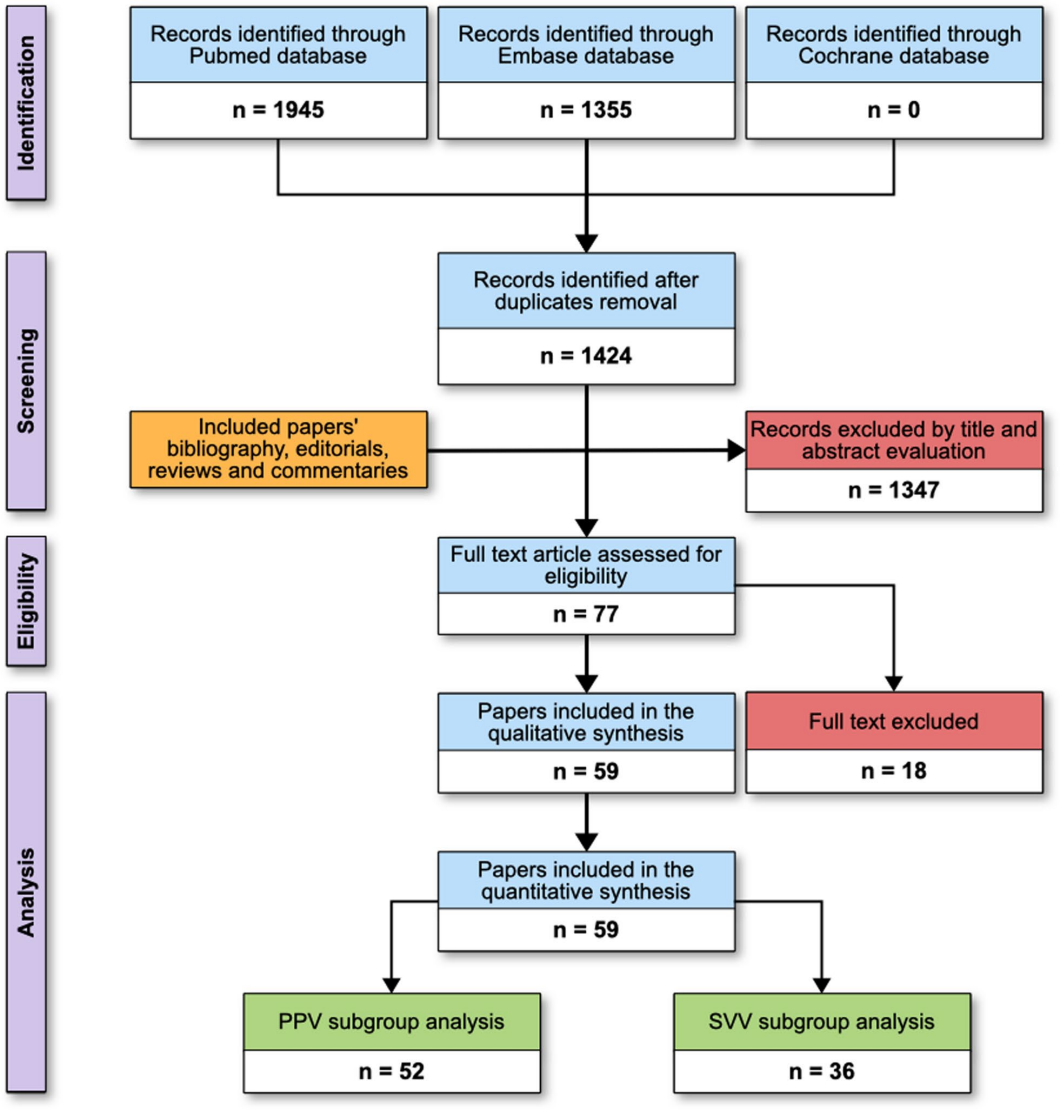
Flow of the studies

The electronic search identified 3,300 potentially relevant titles and 59 full-text manuscripts were finally selected. A detailed description of the selection process is provided in Fig. 1. Overall, the included studies enrolled 2,947 patients with a median age of 61 (55–65), and 59% (46–71) were males. The median number of patients enrolled per study was 40 (26–52), overall receiving 3,870 FCs with a median number of FCs administered of 40 (25–53) for each study and a median of fluid responders of 55% (46–63), ranging from 26.9 [19] to 91.4% [20], and colloids have been used in 41 studies (68.3%) (Table 1).

**Table 1:** Fluid challenge characteristics and hemodynamic monitoring in the included studies

Authors	Year	*n*	FCs	Volume infused (ml)	Infusion time (min)	Fluid type	Monitoring devices	Reference Variable	Responders (%)	Authors	Year	*n*	FCs	Volume infused (ml)	Infusion time (min)	Fluid type	Monitoring devices	Reference Variable	Responders (%)
Hofer C.K., et al. [44]	2005	40	40	10 ml/kg	20	HES 6%	PiCCOplus	SV ≥ 10%	60.0	De Broca B., et al. [28]	2016	60	60	500	10	Saline 0.9%	CardioQ	SV ≥ 15%	62.0
Preisman S., et al. [45]	2005	18	70	250	5–7	Colloids	PiCCO	VTI ≥ 15%	46.0	Biais M., et al. [22]	2017	44	88	250	10	Saline 0.9%	ProAQT	SV ≥ 10%	31.8
Cannesson M., et al. [46]	2007	25	25	500	10	HES 6%	PAC	CI ≥ 15%	60.0	Biais M., et al. [23]	2017	28	28	250	> 10	Saline 0.9%	ProAQT	SV ≥ 10%	57.1
Lee J.-H., et al. [47]	2007	20	20	7 ml/kg	1mL/kg/min	HES 6%	TEE	SVI ≥ 15%	55.0	Biais M., et al. [27]	2017	41	41	250	10	Saline 0.9%	ProAQT	SV ≥ 10%	51.2
Cannesson M., et al. [48]	2008	25	25	500	10	HES 6%	PAC	CI ≥ 15%	68.0	Messina A., et al. [49]	2017	46	46	500	10	Saline 0.9%	MostCare	CI > 15%	41.3
Cannesson M., et al. [50]	2008	25	25	500	> 10	HES 6%	PAC	CI ≥ 15%	64.0	Min J.J., et al. [51]	2017	49	49	6mL/kg	≤10	Saline 0.9%	PAC	NA	42.0
Cannesson M., et al. [52]	2009	25	25	500	10	HES 6%	Mixed	CI ≥ 15%	68.0	Jeong D.M., et al. [53]	2017	79	79	7mL/kg	30	HES 6%	Vigileo/Flotrac	SVI ≥ 10%	37.0
Derichard A., et al. [54]	2009	11	56	200–500	8–15	Colloids	Mixed	CI ≥ 15%	57.1	Min J.J., et al. [55]	2017	40	40	6mL/kg	≤10	HES 6%	NICOM	CI ≥ 12%	47.5
De Waal E., et al. [56]	2009	22	22	10 ml/kg	10	HES 6%	PICCO2	SVI ≥ 12%	83.3	Zlicar M., et al. [35]	2018	56	56	3mL/kg	> 5	HES 6%	LiDCO	nSI ≥ 10%	73.0
Biais M., et al. [57]	2010	27	54	500	10	HES 6%	Vigileo System	SVI ≥ 10%	63.0	Kim D.-H., et al. [36]	2018	53	53	6ml/kg	10	HES 6%	CardioQ	SVI ≥ 15%	75.0
Suheiro K., et al. [58]	2010	30	30	500	30	HES 6%	Vigileo/Flotrac	SVI ≥ 25%	50.0	Weil G., et al. [31]	2019	49	115	250	5	HES 6%	Mixed	SVOD ≥ 15%	62.6
Cannesson M.,et al. [8]	2011	413	413	500	10–20	Colloids	Mixed	CO ≥ 15%	51.0	Joosten A., et al. [21]	2019	57	57	5mL/kg	10	Other colloid	Mixed	COTD ≥ 10%	46.0
Lee J.-H., et al. [59]	2011	49	49	7 ml/kg	7mL/kg/min	HES 6%	TEE	CI > 15%	72.0	Jun J.-H., et al. [34]	2019	38	38	6mL/kg	10	HES 6%	CardioQ	SVI ≥ 10%	63
Høiseth, L., et al. [60]	2011	25	34	250	2–2.5	HES 6%	Mixed	SV ≥ 15%	64.7	Messina A., et al. [26]	2019	40	40	250	10	Ringer’s Lactate	MostCare	SVI > 10%	52.5
Biais M., et al. [61]	2011	35	35	500	10	HES 6%	Vigileo/Flotrac	SV ≥ 15%	57.1	Ali A., et al. [62]	2019	88	88	500	10	Saline 0.9%	Vigileo/Flotrac	SVI ≥ 15%	44.0
Suheiro K., et al.[63]	2011	73	73	500	30	HES 6%	Vigileo/Flotrac	CI ≥ 15%	64.0	Vistisen S.T., et al. [64]	2019	61	122	5mL/kg	10	Saline 0.9%	Vigileo/Flotrac	SV > 10%	56.7
Høiseth, L., et al. [65]	2012	20	22	250	2–2.5	HES 6%	Mixed	SVOD ≥ 15%	31.8	Ali A., et al. [66]	2019	48	48	500	10	Saline 0.9%	Vigileo/Flotrac	SVI ≥ 15%	44.0
Kim S.Y., et al. [67]	2013		66	500	15–20	HES 6%	Mixed	SVI ≥ 12%	63.6	Ali A., et al. [68]	2019	33	66	500	10	Saline 0.9%	Vigileo/Flotrac	SVI ≥ 15%	45.0
Seo H., et al. [69]	2015	39	39	500	20	HES 6%	Vigileo/Flotrac	MAP ≥ \ 15%	44.0	Kimura A., et al. [33]	2021	30	30	250	10	HES 6%	Vigileo/Flotrac	SV/MAP ≥ 10%	57.0
Berger K., et al. [70]	2015	52	52	250	30	HES 6%	Vigileo/Flotrac	SVI ≥ 20	42.3	Watanabe R., et al. [71]	2021	30	30	250	10	HES 6%	Vigileo/Flotrac	SVI ≥ 15%	43.3
Tusman G., et al. [72]	2016	52	52	500	10	Saline 0.9%	PiCCO	CI ≥ 15%	40.0	Kimura A., et al. [29]	2022	30	30	250	10	HES 6%	Vigileo/Flotrac	SV > 10%	67.0
Montenij L.J., et al. [73]	2016	22	22	7mL/kg	15	Saline 0.9%	Vigileo/Flotrac	CO ≥ 15%	40.9	Flick M., et al. [74]	2022	33	33	500	NA	Saline 0.9%	PAC	CI > 15%	39.0
										Shen J., et al. [[24]	2022	80	80	6mL/kg	10	HES 6%	TTE	SVI ≥ 15%	55.0

HES 6%, Hydroxyethylstarch 6%; OD, esophageal doppler; FloTrac/Vigileo/Vigilance/ ThermodilutionVIP + ™, Edwards Lifesciences Co., Irvine, Ca, USA; TTE, Transthoracic echocardiography, *TEE* Transesophageal echocardiography, Pulsioflex/ProAQT®, Maquet, Rastatt, Germany, *PAC* Pulmonary artery catheter; PiCCO/ProAQT/PICCO2, PULSION Medical Systems, MostCare, Pressure Recording Analytical Method, PRAM, Vytech Health®, Padova, Italy; CardioQ™, Deltex Medical Ltd., Chichester (esophageal doppler monitor), UK; NICOM, Non-Invasive Continuous Cardiac Output, Imedex, France; IntelliVue©, Phillips Medical Systems, MA, USA; LiDCO, LiDCO Group PLC, London, UK, *CO* Cardiac output, *CI* Cardiac index, *SV* Stroke volume, *SVI* Stroke volume index, *VTI* Velocity time integral, *R* Responders, *Vol* Volume, *R* Responders, *Vol* Volume, *NA* Not available, *PAC* Pulmonary arterial catheter

Preoperative comorbidities were reported for 2280 patients (76.1%), with cancer diagnoses being the most represented (32.7%). Surgery type was reported for 2932 patients (99.5%), with neurosurgical operations (26.4%) being the most prevalent (Additional file 1: Table S3). Seven studies (11.6%) enrolled only patients undergoing LPS, in 25 (42.4%) the patients received halogenate/opiate anesthesia, while in 23 (39.0%) TIVA and in the remaining 11 studies (18.6%) the type of anesthesia was mixed or unspecified.

Overall, the median (IQR) QUADAS-2 score of the included studies was 9 (7 -10) and 18 studies (30.5%) were classified in the subgroup with the highest risk of bias (Additional file 1: Table S5).

### Overall pooled AUC of PPV and SVV in the included studies

The pooled AUC for the PPV obtained from 52 studies was 0.77 (0.73–0.80), with a mean threshold of 10.8 (10.6–11.0) (*I*^2^ = 92.2%) (Additional file 1: Table S6).

The pooled AUC for the SVV obtained from 36 studies was 0.76 (0.72–0.80), with a mean threshold of 12.1 (11.6–12.7) (*I*^2^ = 88.3%) (Additional file 1: Table S7).

Overall, 19 studies (32.2%) reported the grey zone of PPV or SVV, with a median of 56% (40–62) and 57% (46–83) of patients included in this range of uncertainty, respectively.

### Pooled AUC and grey zone in the different PPV subgroups

1. In the studies enrolling patients with closed chest and abdomen, the pooled AUC for PPV was 0.79 (95%CI 0.73–0.84) for a threshold of 10.9% (10.5–11.2) and a *I*^2^ of 92.7%. (Additional file 1: Figure S1); 7 studies [21–27] reported a median of 61.8% of patients (52–75) included in the grey zone of PPV, with a median low value of 6% (5–8) and a high value of 12% (11–17).
2. In the studies enrolling patients with closed chest and open abdomen, the pooled AUC for PPV was 0.79 (95%CI 0.71–0.88) for a threshold of 11.5% (11.3–11.6) and a *I*^2^ of 88.2%. (Additional file 1: Figure S2); 6 studies [8, 25, 26, 28–32] reported a median of 43.5% (36–50) included in the grey zone of PPV, with a median low value of 7% (5–10) and a high value of 14% (12–25).
3. In the studies enrolling patients with closed abdomen and open chest, the pooled AUC for PPV was 0.69 (95%CI 0.59–0.78) for a threshold of 6.9% (6.7–7.11) and a *I*^2^ of 68.8%. (Additional file 1: Figure S3); 1 study [33] reported 86.0% of patients included in the grey zone of PPV, with a low value of 5% and a high value of 19%.
4. Studies including patients undergoing LPS showed a pooled PPV AUC of 0.74 (95%CI 0.64–0.83), with a pooled threshold of 11.3% (10.6 – 11.9) and a I^2^ of 60.7%. (Additional file 1: Figure S4); 1 study [34] reported 26% of patients included in the grey zone of PPV, and 2 studies [34, 35] a median low value of 6% (6–7) and a high value of 15% (9–21).
5. In studies including patients in prone position, the pooled PPV AUC was 0.78 (95%CI 0.69–0.88), with a pooled threshold of 11.2% (10.9–11.5) and a *I*^2^ of 84.9%. (Additional file 1: Figure S5); 2 studies [36, 37] reported a median of 60.0% (58–62) included in the grey zone of PPV, with a median low value of 6% (5–6) and a high value of 11% (10–11),

### Pooled AUC and grey zone in the different SVV subgroups

1. In the studies enrolling patients with closed chest and abdomen, the pooled AUC for SVV was 0.76 (95%CI 0.69–0.82) for a threshold of 10.7% (10.4–10.9) and a *I*^2^ of 78.4%. (Additional file 1: Figure S6); 1 study [26] reported 88.5% of patients included in the grey zone of SVV, while 2 studies [30, 31] reported a median low value of 5% (3–7) and a high value of 15% (13–16).
2. In the studies enrolling patients with closed chest and open abdomen, the pooled AUC for SVV was 0.79 (95%CI 0.70–0.88) for a threshold of 10.1% (9.8–10.5) and a *I*^2^ of 86.2%. (Additional file 1: Figure S7); 3 studies [28, 30, 31] reported a median of 46.0% (33.0–57.0) included in the grey zone of SVV, with a median low value of 5% (4–6) and a high value of 12% (11–15).
3. In the studies enrolling patients with closed abdomen and open chest, the pooled AUC for SVV was 0.72 (95%CI 0.57–0.87) for a threshold of 10.0% (9.8–10.2) and a *I*^2^ of 85.7%. (Additional file 1: Figure S8); 1 study [33] reported 93.0% of patients included in the grey zone of SVV, with a low value of 5% and a high value of 18%.
4. Studies including patients undergoing LPS showed a pooled SVV AUC of 0.78 (95%CI 0.69–0.87), with a pooled threshold of 10.8% (10.4–11.3) and a *I*^2^ of 64.4%. (Additional file 1: Figure S9); 1 study [34] reported 55.0% of patients included in the grey zone of SVV, while 3 studies [34, 35, 38] reported a median low value of 7% (3–13) and a high value of 13% (6–15).
5. In studies including patients in prone position, the pooled SVV AUC was 0.73 (95%CI 0.64–0.83), with a pooled threshold of 10.2% (9.9–10.4) and a *I*^2^ of 74.9%. (Additional file 1: Figure S10); 1 study [37] reported 66% of patients included in the grey zone of SVV, with a median low value of 6% and a high value of 14%.

Data about pooled ROC and grey zones of the considered subgroups for PPV and SVV are summarized in the Table 2.

**Table 2:** Summary of pooled AUCs and grey zones of PPV and SVV in the considered subgroups

Subgroup	PPV				SVV			
	Pooled AUC	Threshold (%)	Grey zone L	Grey zone H	Pooled AUC	Threshold	Grey zone L	Grey zone H
Closed Chest and Abdomen	0.79	10.9	6	12	0.75	10.7	5	15
Closed Chest and = pen Abdomen	0.79	11.5	7	14	0.79	10.1	5	12
Open Chest and Closed Abdomen	0.69	6.9	5	19	0.72	10.0	5	18
LPS	0.74	11.3	6	15	0.78	10.8	7	13
Prone	0.78	11.2	6	11	0.73	10.2	6	14

Complete data analysis is reported in the Results section. *AUC* Area under receiver operator characteristic curve, *PPV* Pulse pressure variation, *SVV* Stroke volume variation *L* Low value of grey zone, *H* High value of grey zone, *LPS* Laparoscopy

### Metaregression

As shown in Table 3, the pooled AUC for PPV was positively affected by the by Vt ≥ 8 ml/kg (*p* < 0.001) and by the use of colloids for the FC (p < 0.001) in the group of studies with closed chest and abdomen; the Vt ≥ 8 ml/kg (p < 0.001) was also associated to increased AUC in studies enrolling prone patients. The AUCs of these subgroups are reported in the Table 4. The was no effect of any of the considered variables on the AUC of SVV.

**Table 3:** Metaregression of PPV and SVV ROC curves in the considered subgroups of studies

PPV	Closed Abdomen Closed Chest (*N* = 20)	Open Abdomen Closed Chest (*N* = 8)	Closed Abdomen Open Chest (*N* = 9)	Prone (*N* = 9)	Laparoscopy (*N* = 6)
*PEEP (cmH*_*2*_*O)*					
*0*	Ref	Ref	Ref	Ref	Ref
*0–5*	− 0.08 (*p* = 0.39)	− 0.15 (*p* = 0.35)	0.05 (*p* = 0.69)	− 0.09 (*p* = 0.38)	− 0.16 (*p* = 0.31)
> *5*	− 0.20 (*p* = 0.09)	− 0.24 (*p* = 0.19)	NA	NA	− 0.20 (*p* = 0.34)
*VT* > *8 *ml/Kg	**0.20 (*p* < 0.001)**	0.02 (*p* = 0.86)	0.07 (*p* = 0.55)	**0.22 (*p* = 0.03)**	− 0.14 (*p* = 0.31)
*TIVA*	0.00 (*p* = 0.97)	0.14 (*p* = 0.26)	NA	0.06 (*p* = 0.61)	− 0.07 (*p* = 0.65)
*COLLOIDS*	**0.18 (*p* < 0.001)**	− 0.03 (*p* = 0.76)	− 0.06 (*p* = 0.70)	0.11 (*p* = 0.31)	0.03 (*p* = 0.86)
*Volume* > *4 *ml/kg	0.11 (*p* = 0.05)	0.13 (*p* = 0.22)	NA	0.17 (*p* = 0.32)	0.17 (*p* = 0.14)
*Rate ≥ 15 *Min	0.02 (*p* = 0.89)	NA	− 0.07 (*p* = 0.58)	− 0.08 (*p* = 0.63)	− 0.07 (*p* = 0.65)

SVV	Closed abdomen Closed chest (*N* = 9)	Open abdomen Closed chest (*N* = 8)	Closed abdomen Open chest (*N* = 6)	Prone (*N* = 7)	Laparoscopy (*N* = 6)
*PEEP (cmH*_*2*_*O)*					
*0*	Ref	Ref	Ref	Ref	Ref
*0–5*	− 0.06 (*p* = 0.56)	− 0.09 (*p* = 0.38)	0.25 (*p* = 0.29)	− 0.03 (*p* = 0.78)	0.02 (*p* = 0.87)
> *5*	− 0.17 (*p* = 0.18)	− 0.10 (*p* = 0.51)	NA	NA	− 0.22 (*p* = 0.22)
*VT* > *8 *ml/Kg	0.07 (*p* = 0.34)	0.10 (*p* = 0.34)	0.22 (*p* = 0.19)	0.17 (*p* = 0.07))	0.02 (*p* = 0.91)
*TIVA*	− 0.00 (*p* = 0.95)	0.14 (*p* = 0.24)	− 0.25 (*p* = 0.29)	0.14 (*p* = 0.15)	− 0.23 (*p* = 0.09)
*COLLOIDS*	0.12 (*p* = 0.06)	− 0.01 (*p* = 0.91)	NA	0.13 (*p* = 0.21)	− 0.17 (*p* = 0.07)
*Volume* > *4 *ml/kg	0.12 (*p* = 0.08)	0.04 (*p* = 0.65)	NA	0.11 (*p* = 0.33)	0.01 (*p* = 0.91)
*Rate* ≥ *15 *Min	0.02 (*p* = 0.82)	NA	− 0.22 (*p* = 0.19)	0.00 (*p* = 0.98)	− 0.23 (*p* = 0.09)

Bold variables indicates statistically significant values

*PPV* Pulse pressure variation, *SVV* Stroke volume variation, *ROC* Receiver operator characteristic curve, *PEEP* Positive end-expiratory pressure, *VT* Tidal volume, *TIVA* Total intravenous anesthesia, *NA* Non-applicable for collinearity; ref, reference, ml/Kg Milliliters per kilogram, *min* Minutes. Data are expressed as coefficient of metaregression (*p* value); ref, reference

**Table 4:** Difference in the AUCs of the subgroups positively affected by the variables analyzed in the metaregression

Closed Chest and Closed Abdomen				Prone patients	
AUC of PPV		AUC of PPV		AUC of PPV	
VT > 8 ml/Kg	VT ≤ 8 ml/Kg	Colloids YES	Colloids NO	VT > 8 ml/Kg	VT ≤ 8 ml/Kg
0.88 (0.82–0.93)	0.69 (0.65 – 0.74)	0.86 (0.80–0.92)	0.70 (0.65 – 0.75)	0.85 (0.76–0.94)	0.64 (0.54 – 0.74)

*PPV* Pulse pressure variation, *SVV* Stroke volume variation, *AUC* Area under receiver operator characteristic curve, *PEEP* Positive end-expiratory pressure, *VT* Tidal volume, *TIVA* Total intravenous anesthesia, *NA* Non-applicable for collinearity, *ref* Reference, ml/Kg Milliliters per kilogram, *min* Minutes

Data are expressed as coefficient of metaregression (p value); ref, reference

## Discussion

This systematic review and metanalysis evaluated the PPV and SVV performance in different surgical settings, updating previous papers and focusing on the role of potential factors that may be associated with better performance of the indices for predicting fluid responsiveness. Our data may be summarized as follows: (1) the overall performance of PPV and SVV in operating room in predicting fluid responsiveness is moderate, ranging close to an AUC of 0.80 only in non-LPS surgery, with closed chest, suggesting caution in the interpretation of this indices; (2) overall, the best threshold of PPV is 11%, while for the SVV is 10%. However, the minority of the studies reporting the grey zone showed that the majority of patients are patients included in this range of uncertainty, respectively; (3) a high Vt and the choice of colloids, may impact positively on PPV performance, especially among patients with closed chest and abdomen, or in prone position.

PPV and SVV have been widely investigated as indices to guide fluid administration, but also as targets of a goal-directed therapy [22]. In the operating room, most of the validity criteria affecting PPV and SVV reliability (such as low tidal volume, heart rate/respiratory rate ratio < 3.6, presence of spontaneous breathing activity, low respiratory compliance, right ventricle dysfunction) occur less frequently compared to critically ill patients. This would, in principle, improve their performance as tests for fluid responsiveness. The role of Vt has also been extensively investigated. In 2009 Marik et al. reported on an AUC of 0.93 (95% CI, 0.92–0.94) for the PPV in a small subgroup of surgical studies adopting a mean Vt > 8 ml/kg [39]. In 2011 Zhang et al. reported an AUC of 0.94 (95% CI, 0.907–0.945) for the SVV in 8 surgical studies [40], decreasing to 0.84 by excluding only one study on 20 patients [41]. Similarly, Messina et al. in 2018 reported an AUC of 0.86 for PPV (10 studies) and of 0.87 for SVV (16 studies) in surgical trials with a mean Vt of 8 ml/kg [11]. The metaregression showed that the only AUCs of the PPV in the subgroups of patients with closed chest and abdomen or in prone position were positively affected by the intraoperative use of a Vt ≥ 8 ml/kg. Although the use of an intraoperative lung-protective ventilation strategy is associated with a better outcome [9] and is now suggested as standard practice in the operating room [10], this limits the assessment of fluid responsiveness in surgical patients by means of dynamic indices and, not surprisingly, pooling data from recent studies show an overall worse performance of PPV, as compared to the past.

Accordingly to the reduced Vt, also the thresholds of PPV and SVV should be reconsidered. Our results suggest a best pooled threshold for PPV (11%) and SVV (10%) both lower than 13% proposed in the past [8]. However, these thresholds derived from ROC curve analysis may be scarcely useful in clinical practice, since it often falls within the grey zone of uncertainty. Considering the different subgroups, our results show that the range of PPV and SVV values included between the lowest 5–7% and the highest of 12–19%, should be considered with caution, suggesting the use of other functional hemodynamic tests in surgical patients for enhancing the reliability of these dynamic indices [17].

The metaregression showed that the AUC of the PPV in the subgroups of patients with closed chest and abdomen was improved by the use of colloids (*p* < 0.001) and, potentially, by a FC volume > 4 ml/kg (borderline effect; *p* = 0.05). For the SVV, these two variables showed also borderline effects (*p* = 0.06 and *p* = 0.08, respectively). Recently, it has been demonstrated that at least 4 ml/kg should be infused to effectively challenge cardiac preload [14, 42]. Accordingly, reducing FC volume would impact on the identification of fluid responders and, in turn, on AUC magnitude. Colloids are still adopted in the operating room, and their different persistence in the intravascular space may affect fluid responsiveness especially when the time of evaluation of FC is prolonged above 10 min (when the effect of a crystalloid FC fades[13]).

### Strenghts and limitations

To the best of our knowledge, this is the most updated and largest metanalysis on PPV and SVV use in the operating room. Our approach considered the physiologic characteristics of the surgical patients and not the specific type of surgery. This implies that the results may be applied to different settings (i.e. the subgroup with closed chest and abdomen may include neurosurgery, vascular non-abdominal surgery and otolaryngology surgery). Moreover, the meta-regression analysis enhanced specific variables potentially affecting PPV and SVV reliability.

Regarding the limitations, despite the minority of the studies (30.5%) were classified in the subgroup with the highest risk of bias, the QUADAS-2 score, however, as any other bias score, would not perfectly fit to the design of the included studies, and it has been adapted by the authors in some domain, considering clinical of physiological variables potentially affecting FC outcome and, hence, ROC curve analysis.

Moreover, the heterogeneity of the AUCs obtained from of the analyzing data ranged from 43.5 to 88.2%, implying a significant variability in the population enrolled and data presentation. Again, this is, unfortunately, a quite common problem in the field of hemodynamic for either critically ill and surgical patients as previously shown in other papers [17, 43]. Overall, data obtained from the meta-regression should be considered with caution, due to the small number of studies included in some subgroup.

This is not a meta-analysis based on individual data and the assessment of fluid responsiveness has been evaluated by different hemodynamic tools, including echocardiography, calibrated and uncalibrated machines.

The authors state some discrepancies between the final literature search, focused on PPV and SVV use in studies assessing the performance of these variables in predicting fluid responsiveness, and the original PROSPERO registration, which includes also randomized-controlled studies adopting PVV and SVV in the context of perioperative hemodynamic optimization. After an initial screening, it was clear that these studies should have been not included in the literature search, and the string has been modified, accordingly.

## Conclusions

The overall performance of PPV and SVV in operating room in predicting fluid responsiveness is moderate, ranging close to an AUC of 0.80 only for some subgroups of surgical patients, with a best threshold of 11% and 10%, respectively. Considering the different subgroups, the grey zone of these dynamic indices (from 5 to 7% and to 12 to 19%) is wide and should be carefully considered during the assessment of fluid responsiveness. A high Vt and the choice of colloids for the FC, may impact positively on the performance of the dynamic indices, especially among patients with closed chest and abdomen, or in prone position.

### Supplementary Information

**Additional file 1.** Supplementray Tables and Figures.

## Data Availability

The datasets used and/or analyzed during the current study are available from the corresponding author on reasonable request.

## Abbreviations

FC: Fluid challenge
MV: Mechanical ventilation
SVV: Stroke volume variation
PPV: Pulse pressure variation
SV: Stroke volume
AUC: Area under the curve (AUC) of pooled receiving operating characteristics (ROC) curve
LPS: Laparoscopy
Vt: Tidal volume
