# Supplementary material for: Reliability of pulse pressure and stroke volume variation in assessing fluid responsiveness in the operating room: a metanalysis and a metaregression
Source: Crit Care. 2023 Nov 8;27:431. doi: 10.1186/s13054-023-04706-0 (PMC10631038; doi:10.1186/s13054-023-04706-0)
Supplement: Supplementary file 1 — Additional file 1. Supplementray Tables and Figures. [file 13054_2023_4706_MOESM1_ESM.docx]

**Reliability of pulse pressure and stroke volume variation in assessing fluid responsiveness in the operating room: a metanalysis and a metaregression.**

**Running title: PPV and SVV reliability in surgical settings**

Antonio Messina^1,2^; Mariagiovanna Caporale^3^; Lorenzo Calabrò^1^; Giulia Lionetti^1^; Daniele Bono^1^; Guia Margherita Matronola^1^; Andrea Brunati^1^; Luciano Frassanito^3^; Emanuela Morenghi^1,2^; Massimo Antonelli^3^; Michelle Chew^4^; Maurizio Cecconi^1,2^.

^1^IRCCS Humanitas Research Hospital, Via Manzoni 56, 20089 Rozzano - Milano, Italy.

^2^Department of Biomedical Sciences, Humanitas University, Pieve Emanuele - Milano, Italy.

^3^Department of Anesthesia and Intensive Care, Fondazione Policlinico Universitario ‘A. Gemelli’ IRCCS, Università Cattolica del Sacro Cuore, Rome, Italy.

^4^Department of Anaesthesia and Intensive Care, Biomedical and Clinical Sciences, Linköping, Linköping, University, Sweden.

Corresponding author:

Antonio Messina

Department of Anaesthesia and Intensive Care Medicine - IRCCS Humanitas Research Hospital

Via Alessandro Manzoni, 5620089 – Rozzano, Milano - Italy

Email: antonio.messina@humanitas.it

Tel: +39(0)2 8224 1

**Supplementary materials**

**Supplemental Table 1. PRISMA DTA**

| **Section/topic** | **#** | **PRISMA-DTA Checklist Item** | **Reported on page #** |
| --- | --- | --- | --- |
| **TITLE / ABSTRACT** | | |  |
| Title | 1 | Identify the report as a systematic review (+/- meta-analysis) of diagnostic test accuracy (DTA) studies. | 1 |
| Abstract | 2 | Abstract: See PRISMA-DTA for abstracts. | 2 |
| **INTRODUCTION** | | |  |
| Rationale | 3 | Describe the rationale for the review in the context of what is already known. | 4-5 |
| Clinical role of index test | D1 | State the scientific and clinical background, including the intended use and clinical role of the index test, and if applicable, the rationale for minimally acceptable test accuracy (or minimum difference in accuracy for comparative design). | 4-5 |
| Objectives | 4 | Provide an explicit statement of question(s) being addressed in terms of participants, index test(s), and target condition(s). | 4-5 |
| **METHODS** | | |  |
| Protocol and registration | 5 | Indicate if a review protocol exists, if and where it can be accessed (e.g., Web address), and, if available, provide registration information including registration number. | NA |
| Eligibility criteria | 6 | Specify study characteristics (participants, setting, index test(s), reference standard(s), target condition(s), and study design) and report characteristics (e.g., years considered, language, publication status) used as criteria for eligibility, giving rationale. | 6 |
| Information sources | 7 | Describe all information sources (e.g., databases with dates of coverage, contact with study authors to identify additional studies) in the search and date last searched. | 6-7 |
| Search | 8 | Present full search strategies for all electronic databases and other sources searched, including any limits used, such that they could be repeated. | 6-7 |
| Study selection | 9 | State the process for selecting studies (i.e., screening, eligibility, included in systematic review, and, if applicable, included in the meta-analysis). | 6-7 |
| Data collection process | 10 | Describe method of data extraction from reports (e.g., piloted forms, independently, in duplicate) and any processes for obtaining and confirming data from investigators. | 6-7 |
| Definitions for data extraction | 11 | Provide definitions used in data extraction and classifications of target condition(s), index test(s), reference standard(s) and other characteristics (e.g. study design, clinical setting). | 7 |
| Risk of bias and applicability | 12 | Describe methods used for assessing risk of bias in individual studies and concerns regarding the applicability to the review question. | 7 |
| Diagnostic accuracy measures | 13 | State the principal diagnostic accuracy measure(s) reported (e.g. sensitivity, specificity) and state the unit of assessment (e.g. per-patient, per-lesion). | 7-8 |
| Synthesis of results | 14 | Describe methods of handling data, combining results of studies and describing variability between studies. This could include, but is not limited to: a) handling of multiple definitions of target condition. b) handling of multiple thresholds of test positivity, c) handling multiple index test readers, d) handling of indeterminate test results, e) grouping and comparing tests, f) handling of different reference standards | 7-8 |

| **Section/topic** | **#** | **PRISMA-DTA Checklist Item** | **Reported on page #** |
| --- | --- | --- | --- |
| Meta-analysis | D2 | Report the statistical methods used for meta-analyses, if performed. | 9-11 |
| Additional analyses | 16 | Describe methods of additional analyses (e.g., sensitivity or subgroup analyses, meta-regression), if done, indicating which were pre-specified. | 9-11 |
| **RESULTS** | | |  |
| Study selection | 17 | Provide numbers of studies screened, assessed for eligibility, included in the review (and included in meta-analysis, if applicable) with reasons for exclusions at each stage, ideally with a flow diagram. | 9 |
| Study characteristics | 18 | For each included study provide citations and present key characteristics including: a) participant characteristics (presentation, prior testing), b) clinical setting, c) study design, d) target condition definition, e) index test, f) reference standard, g) sample size, h) funding sources | 9 |
| Risk of bias and applicability | 19 | Present evaluation of risk of bias and concerns regarding applicability for each study. | 9 |
| Results of individual studies | 20 | For each analysis in each study (e.g. unique combination of index test, reference standard, and positivity threshold) report 2x2 data (TP, FP, FN, TN) with estimates of diagnostic accuracy and confidence intervals, ideally with a forest or receiver operator characteristic (ROC) plot. | 9 |
| Synthesis of results | 21 | Describe test accuracy, including variability; if meta-analysis was done, include results and confidence intervals. | 9-11 |
| Additional analysis | 23 | Give results of additional analyses, if done (e.g., sensitivity or subgroup analyses, meta-regression; analysis of index test: failure rates, proportion of inconclusive results, adverse events). | 9-11 |
| **DISCUSSION** | | |  |
| Summary of evidence | 24 | Summarize the main findings including the strength of evidence. | 13-14 |
| Limitations | 25 | Discuss limitations from included studies (e.g. risk of bias and concerns regarding applicability) and from the review process (e.g. incomplete retrieval of identified research). | 14-15 |
| Conclusions | 26 | Provide a general interpretation of the results in the context of other evidence. Discuss implications for future research and clinical practice (e.g. the intended use and clinical role of the index test). | 16 |
| **FUNDING** | | |  |
| Funding | 27 | For the systematic review, describe the sources of funding and other support and the role of the funders. | 18 |

*Adapted From:*  McInnes MDF, Moher D, Thombs BD, McGrath TA, Bossuyt PM, The PRISMA-DTA Group (2018). Preferred Reporting Items for a Systematic Review and Meta-analysis of Diagnostic Test Accuracy Studies: The PRISMA-DTA Statement. JAMA. 2018 Jan 23;319(4):388-396. doi: 10.1001/jama.2017.19163.

**Supplemental Table 2. Full search criteria**

| EMBASE/MEDLINE | Query Results | Results |
| --- | --- | --- |
| Search | 'pulse pressure variation'/exp OR 'stroke volume variation' OR 'fluid responsiveness' | 3,031 |
| Filters | AND 'human'/de AND ([adult]/lim OR [aged]/lim OR [middle aged]/lim OR [very elderly]/lim OR [young adult]/lim | 1,355 |
| PUBMED | Query Results | Results |
| Search | ((((pulse pressure variation[Title/Abstract]) OR (stroke volume variation[Title/Abstract])) OR (fluid responsiveness[Title/Abstract])) AND (surgery[Title/Abstract])) OR (surgical patients[Title/Abstract]) | 24,185 |
| Filters | Clinical Trial, Humans, English, Adult: 19+ years, Young Adult: 19-24 years, Adult: 19-44 years, Middle Aged + Aged: 45+ years, Middle Aged: 45-64 years, Aged: 65+ years, 80 and over: 80+ years. | 1,945 |

**Supplemental Table 3. Characteristics of included patients: comorbidities and type of surgery**

| General characteristics | n = 2,997 (IQR) |
| --- | --- |
| Age (year) | 62 (55.4 – 65) |
| Male (%) | 59 (46.3 – 72) |
| Weight (kg) | 68.8 (62.5 – 77) |
| Height (cm) | 167 (163.5 – 170) |
| BMI | 25 (24 – 27) |
| Comorbidities | **n = 2,280 (%)** |
| Hypertension | 646 (28.3) |
| Coronary Artery Disease | 376 (16.5) |
| Diabetes Mellitus | 259 (11.4) |
| Chronic Obstructive Pulmonary Disease | 112 (4.9) |
| Peripheral Vascular Disease | 64 (2.8) |
| Stroke | 42 (1.8) |
| Heart Failure | 35 (1.5) |
| Cancer | 746 (32.7) |
| Type of Surgery | **n = 2,982 (%)** |
| General (unspecified) | 577 (19.3) |
| Vascular | 285 (9.6) |
| Orthopedic | 2 (0.1) |
| Neurosurgery | 787 (26.4) |
| Cardiac | 682 (22.9) |
| Urological | 136 (4.6) |
| Gynecological | 177 (5.9) |
| Thoracic | 331 (11.1) |
| Other | 5 (0.2) |
| N, number of patients; %DR, percentage of data reported in the studies. | |

**Supplemental Table 4 –** Definition of potential bias for the enrolled studies.

|  |  | **CRITERIA** | **LOW RISK** | **UNCLEAR RISK** | **HIGH RISK** |
| --- | --- | --- | --- | --- | --- |
| Patient selection | Risk of Bias | Patients were consecutively included | Consecutive inclusion stated | Period of enrolment indicated and consecutive inclusion not indicated | Non-consecutive inclusion stated and no period of enrolment indicated |
|  |  | Inappropriate exclusions avoided | No inappropriate exclusions | NA | Inappropriate exclusions |
|  | Applicability concerns | Are there concerns that the included patients do not match the review question? | All the others | Beta-Blockers  (> 25% of entolled patients) | Severe cardiac dysfunction  (> 25% of entolled patients)  Mean/median age >75 yo |
| Index test | Risk of Bias | Threshold used to define volume responsiveness pre-specified | Defined | NA | Undefined |
|  | Applicability concerns | Are there concerns that the included patients do not match the review question? | All the others | Infusion FC time  from 10 to 20 minutes  and FC < 4 ml/kg | Infusion FC time > 20 minutes and FC < 4 ml/kg |
| Reference Standard | Risk of Bias | The reliability of the device used in the study widely validated | Echocardiography by experts; PAC; calibrated tool, uncalibrated tool. | Mixed devices | Bioimpedance – Bioreactance  Echocardiography by non experts |
|  | Applicability concerns | Number of FC / patient | One FC for each enrolled patients |  | Multiple FCs |
| Flow and Timing | Risk of Bias | An appropriate interval between volume expansion and effect analysis | ≤ 5 minutes | Undefined | > 5 minutes |
|  |  | All patients were included in the analysis. | All included | NA | Not all included |

The risk of bias of the enrolled studies was defined by The QUADAS 2 score, by two experts. For each domain the risk was judged as “low,” “high,” or “unclear.” If the answers to all signaling questions for a domain were “yes,” then risk of bias was judged low. If any signaling question was answered “no,” then potential bias was assessed by used the reported criteria. The cumulative risk of bias of each criterion was defined by the highest risk of bias reported. NA, not applicable; OR, operating room; PAC, pulmonary artery catheter; FC, fluid challenge.

**Supplemental Table 5 – QUADAS-2 score assessment of the included studies**

|  |  | **Patient selection** | | **Index test** | | **Reference Standard** | | **Flow and timing def.** | **Total** |
| --- | --- | --- | --- | --- | --- | --- | --- | --- | --- |
| **Author Names** | **Publication Year** | **Risk of Bias** | **Applicability concerns** | **Risk of Bias** | **Applicability concerns** | **Risk of Bias** | **Applicability concerns** |  |  |
| Høiseth L.Ø. et al | 2011 | 3 | 1 | 1 | 1 | 2 | 3 | 3 | 14 |
| Høiseth L.Ø. et al | 2012 | 3 | 1 | 1 | 1 | 2 | 3 | 3 | 14 |
| Chin J.H. et al | 2013 | 2 | 1 | 1 | 1 | 2 | 1 | 1 | 9 |
| Weil G. et al | 2019 | 1 | 1 | 1 | 1 | 2 | 3 | 1 | 10 |
| Cannesson M. et al | 2008 | 1 | 2 | 1 | 1 | 1 | 1 | 2 | 9 |
| Cannesson M. et al | 2009 | 1 | 2 | 1 | 1 | 2 | 1 | 1 | 9 |
| Biais M. et al | 2011 | 3 | 2 | 1 | 1 | 1 | 1 | 1 | 10 |
| Tusman G. et al | 2016 | 3 | 1 | 1 | 1 | 1 | 1 | 2 | 10 |
| Jeong D.M. et al | 2017 | 2 | 1 | 1 | 1 | 1 | 1 | 1 | 8 |
| Joosten A. et al | 2019 | 2 | 2 | 1 | 1 | 2 | 1 | 2 | 11 |
| Messina A. et al | 2020 | 1 | 1 | 1 | 2 | 1 | 1 | 2 | 9 |
| Weil G. et al | 2020 | 3 | 1 | 1 | 1 | 1 | 3 | 1 | 11 |
| Kimura A. et al | 2021 | 3 | 1 | 1 | 2 | 1 | 1 | 2 | 11 |
| Cannesson M. et al | 2007 | 1 | 1 | 1 | 1 | 1 | 1 | 1 | 7 |
| Cannesson M.. et al | 2011 | 3 | 1 | 1 | 1 | 2 | 1 | 1 | 10 |
| Biais M. et al | 2017 | 3 | 1 | 1 | 2 | 1 | 1 | 1 | 10 |
| Biais M. et al | 2017 | 3 | 1 | 1 | 2 | 1 | 2 | 2 | 12 |
| Fu Q. et al | 2015 | 3 | 1 | 1 | 1 | 1 | 1 | 1 | 9 |
| Jun J.-H. et al | 2019 | 1 | 1 | 1 | 1 | 1 | 1 | 1 | 7 |
| Watanabe R. et al | 2021 | 3 | 1 | 1 | 2 | 1 | 1 | 1 | 10 |
| Shen J. et al | 2022 | 1 | 1 | 1 | 1 | 1 | 1 | 3 | 9 |
| Preisman S. et al | 2005 | 3 | 1 | 1 | 1 | 1 | 3 | 1 | 11 |
| Lee J.-H. et al | 2007 | 3 | 1 | 1 | 1 | 1 | 1 | 3 | 11 |
| Cannesson M. et al | 2008 | 1 | 2 | 1 | 1 | 1 | 1 | 1 | 8 |
| Derichard A. et al | 2009 | 3 | 1 | 1 | 1 | 2 | 3 | 1 | 12 |
| Biais M. et al | 2010 | 1 | 1 | 1 | 1 | 1 | 3 | 1 | 9 |
| Nordström J. et al | 2013 | 2 | 1 | 1 | 1 | 2 | 3 | 1 | 11 |
| Yang S.-Y. et al | 2013 | 3 | 1 | 1 | 1 | 1 | 3 | 1 | 11 |
| Guinot P.-G. et al | 2014 | 3 | 1 | 1 | 1 | 1 | 1 | 1 | 9 |
| Guinot P.-G. et al | 2014 | 3 | 1 | 1 | 1 | 1 | 1 | 1 | 9 |
| de Courson H. et al | 2020 | 3 | 1 | 1 | 2 | 1 | 3 | 1 | 12 |
| Hofer C.K. et al | 2005 | 3 | 1 | 1 | 1 | 1 | 1 | 3 | 11 |
| Lee J.-H. et al | 2011 | 3 | 1 | 1 | 1 | 1 | 1 | 3 | 11 |
| Messina A. et al | 2017 | 1 | 1 | 1 | 1 | 1 | 1 | 2 | 8 |
| Min J.J. et al | 2017 | 2 | 1 | 1 | 1 | 1 | 1 | 2 | 9 |
| Messina A. et al | 2019 | 1 | 1 | 1 | 2 | 1 | 1 | 2 | 9 |
| Messina A. et al | 2021 | 1 | 1 | 1 | 1 | 1 | 1 | 3 | 9 |
| Choi K.-H. et al | 2021 | 3 | 1 | 1 | 1 | 1 | 1 | 2 | 10 |
| Montenij L.J. et al | 2016 | 3 | 1 | 1 | 1 | 1 | 1 | 3 | 11 |
| Min J.J. et al | 2017 | 2 | 1 | 1 | 1 | 1 | 1 | 2 | 9 |
| Zlicar M. et al | 2018 | 2 | 1 | 1 | 1 | 1 | 1 | 1 | 8 |
| Ali A. et al | 2019 | 1 | 1 | 1 | 1 | 1 | 1 | 3 | 9 |
| Vistisen S.T. et al | 2019 | 1 | 2 | 1 | 1 | 1 | 3 | 3 | 12 |
| Bubenek-Turconi Ş.-I. et al | 2020 | 1 | 1 | 1 | 1 | 1 | 3 | 2 | 10 |
| Kimura A. et al | 2022 | 3 | 1 | 1 | 2 | 1 | 1 | 1 | 10 |
| Flick M. et al | 2022 | 2 | 1 | 1 | 1 | 1 | 1 | 1 | 8 |
| Kim S.Y. et al | 2013 | 3 | 1 | 1 | 1 | 2 | 1 | 3 | 12 |
| Seo H. et al | 2015 | 1 | 1 | 1 | 1 | 1 | 1 | 2 | 8 |
| De Broca B. et al | 2016 | 2 | 2 | 1 | 1 | 1 | 1 | 1 | 9 |
| Lee C.-T. et al | 2020 | 3 | 1 | 1 | 1 | 1 | 1 | 2 | 10 |
| Berger K. et al | 2015 | 3 | 1 | 1 | 3 | 1 | 1 | 2 | 12 |
| Ali A. et al | 2019 | 1 | 1 | 1 | 1 | 1 | 1 | 1 | 7 |
| Ali A. et al | 2019 | 1 | 1 | 1 | 1 | 1 | 3 | 2 | 10 |
| Kim D.-H. et al | 2018 | 3 | 1 | 1 | 1 | 1 | 1 | 2 | 10 |
| Biais M. et al | 2017 | 2 | 1 | 1 | 2 | 1 | 1 | 1 | 9 |
| Koichi Suehiro et al | 2011 | 3 | 1 | 1 | 1 | 1 | 1 | 1 | 9 |
| Qiang Fu* et al | 2014 | 3 | 1 | 1 | 1 | 1 | 1 | 1 | 9 |
| Koichi Suehiro et al | 2010 | 3 | 1 | 1 | 1 | 1 | 1 | 1 | 9 |
| Eric E. C. de Waal et al | 2009 | 2 | 1 | 1 | 1 | 1 | 1 | 1 | 8 |

**Supplemental Table 6. Pooled AUC of PPV**

. meta summarize , from(0.5)

Effect-size label: Effect size Effect size: auc1value

Std. err.: se_auc1 Study label: label_s

Meta-analysis summary Number of studies = 52

Random-effects model Heterogeneity:

Method: REML tau2 = 0.0125

I2 (%) = 92.24

H2 = 12.88

---------------------------------------------------------------------------

Study | Effect size [95% conf. interval] % weight

-------------------------+-------------------------------------------------

Ali A., 2019 | 0.770 0.647 0.893 1.96

Cannesson M., 2008 | 0.923 0.864 0.982 2.40

de Courson H., 2020 | 0.637 0.635 0.639 2.58

Ali A., 2019 | 0.790 0.701 0.879 2.21

Lee J.-H., 2007 | 0.909 0.765 1.053 1.80

Cannesson M., 2008 | 0.941 0.855 1.027 2.24

Cannesson M., 2009 | 0.857 0.774 0.940 2.26

Kim D.-H., 2018 | 0.781 0.664 0.898 2.01

Weil G., 2020 | 0.780 0.657 0.903 1.96

Cannesson M., 2011 | 0.890 0.860 0.920 2.53

Flick M., 2022 | 0.710 0.533 0.887 1.56

Biais M., 2011 | 0.957 0.923 0.991 2.52

Biais M., 2017 | 0.720 0.543 0.897 1.56

De Broca B., 2016 | 0.650 0.522 0.778 1.92

Messina A., 2020 | 0.630 0.458 0.802 1.59

Jun J.-H., 2019 | 0.850 0.727 0.973 1.96

Cannesson M., 2007 | 0.847 0.764 0.930 2.26

Chin J.H., 2013 | 0.870 0.752 0.988 2.00

Zlicar M., 2018 | 0.670 0.516 0.824 1.73

Messina A., 2019 | 0.680 0.508 0.852 1.59

Hofer C.K., 2005 | 0.808 0.669 0.947 1.84

Derichard A., 2009 | 0.960 0.822 1.098 1.85

Ali A., 2019 | 0.683 0.518 0.848 1.64

Seo H., 2015 | 0.671 0.501 0.841 1.60

Høiseth L.Ø., 2011 | 0.670 0.507 0.833 1.66

Weil G., 2019 | 0.670 0.572 0.768 2.14

Vistisen S.T., 2019 | 0.640 0.458 0.822 1.52

Høiseth L.Ø., 2012 | 0.530 0.313 0.747 1.30

Min J.J., 2017 | 0.840 0.717 0.963 1.96

Shen J., 2022 | 0.846 0.763 0.929 2.26

Preisman S., 2005 | 0.950 0.896 1.004 2.43

Biais M., 2017 | 0.750 0.602 0.898 1.77

Nordström J., 2013 | 0.660 0.527 0.793 1.88

Joosten A., 2019 | 0.680 0.552 0.808 1.92

Kimura A., 2022 | 0.670 0.498 0.842 1.59

Watanabe R., 2021 | 0.550 0.366 0.734 1.51

Biais M., 2017 | 0.650 0.527 0.773 1.96

Guinot P.-G., 2014 | 0.710 0.577 0.843 1.88

Tusman G., 2016 | 0.680 0.601 0.759 2.28

Lee C.-T., 2020 | 0.710 0.567 0.853 1.81

Messina A., 2021 | 0.950 0.901 0.999 2.45

Biais M., 2010 | 0.959 0.865 1.053 2.18

Messina A., 2017 | 0.800 0.662 0.938 1.85

Yang S.-Y., 2013 | 0.969 0.926 1.012 2.48

Lee J.-H., 2011 | 0.524 0.286 0.762 1.18

Min J.J., 2017 | 0.760 0.632 0.888 1.92

Lee J.-H., 2011 | 0.857 0.714 1.000 1.80

Eric E. C. de Waal, 2009 | 0.884 0.751 1.017 1.88

Jeong D.M., 2017 | 0.630 0.522 0.738 2.07

Vistisen S.T., 2019 | 0.560 0.402 0.718 1.70

Choi K.-H., 2021 | 0.650 0.473 0.827 1.56

Kimura A., 2021 | 0.560 0.378 0.742 1.52

-------------------------+-------------------------------------------------

theta | 0.770 0.735 0.805

---------------------------------------------------------------------------

Test of theta = 0: z = 42.98 Prob > |z| = 0.0000

Test of homogeneity: Q = chi2(51) = 1500.99 Prob > Q = 0.0000

. mean threshold ppv

Mean estimation Number of obs = 2,219

---------------------------------------------------------------

| Mean Std. err. [95% conf. interval]

--------------+------------------------------------------------

threshold1ppv | 10.85052 .0835753 10.68662 11.01441

---------------------------------------------------------------

**Supplemental Tale 7. Pooled AUC of SVV**

Meta-analysis summary Number of studies = 36

Random-effects model Heterogeneity:

Method: REML tau2 = 0.0110

I2 (%) = 88.37

H2 = 8.60

----------------------------------------------------------------------------

Study | Effect size [95% conf. interval] % weight

--------------------------+-------------------------------------------------

Hofer C.K., 2005 | 0.823 0.679 0.967 2.61

Preisman S., 2005 | 0.870 0.786 0.954 3.33

Cannesson M., 2009 | 0.871 0.787 0.955 3.33

Derichard A., 2009 | 0.950 0.778 1.122 2.28

Eric E. C. de Waal, 2009 | 0.911 0.798 1.024 2.98

Biais M., 2010 | 0.938 0.831 1.045 3.05

Suehiro K., 2010 | 0.900 0.810 0.990 3.27

Suehiro K., 2011 | 0.776 0.598 0.954 2.22

Fu Q., 2012 | 0.862 0.763 0.961 3.15

Høiseth L.Ø., 2012 | 0.740 0.548 0.932 2.07

Chin J.H., 2013 | 0.810 0.682 0.938 2.80

Kim S.Y., 2013 | 0.695 0.570 0.820 2.84

Nordström J., 2013 | 0.720 0.607 0.833 2.98

Guinot P.-G., 2014 | 0.890 0.787 0.993 3.10

Guinot P.-G., 2014 | 0.920 0.841 0.999 3.39

Qiang Fu*, 2014 | 0.507 0.297 0.717 1.90

Berger K., 2015 | 0.760 0.543 0.977 1.84

Seo H., 2015 | 0.595 0.436 0.754 2.43

De Broca B., 2016 | 0.800 0.682 0.918 2.92

Montenij L.J., 2016 | 0.700 0.478 0.922 1.80

Jeong D.M., 2017 | 0.530 0.417 0.643 2.98

Min J.J., 2017 | 0.790 0.657 0.923 2.74

Zlicar M., 2018 | 0.800 0.670 0.930 2.78

Ali A., 2019 | 0.709 0.548 0.870 2.41

Ali A., 2019 | 0.770 0.647 0.893 2.86

Jun J.-H., 2019 | 0.770 0.632 0.908 2.68

Messina A., 2019 | 0.680 0.513 0.847 2.33

Weil G., 2019 | 0.570 0.476 0.664 3.22

Bubenek-Turconi Ş.-I., 2020 0.890 0.851 0.929 3.75

Lee C.-T., 2020 | 0.720 0.572 0.868 2.56

Messina A., 2020 | 0.610 0.556 0.664 3.64

de Courson H., 2020 | 0.649 0.647 0.651 3.89

Choi K.-H., 2021 | 0.640 0.458 0.822 2.17

Messina A., 2021 | 0.710 0.616 0.804 3.22

Watanabe R., 2021 | 0.611 0.431 0.791 2.20

Kimura A., 2022 | 0.660 0.488 0.832 2.28

--------------------------+-------------------------------------------------

theta | 0.761 0.720 0.801

----------------------------------------------------------------------------

Test of theta = 0: z = 36.85 Prob > |z| = 0.0000

Test of homogeneity: Q = chi2(35) = 421.87 Prob > Q = 0.0000

Mean estimation Number of obs = 1,359

---------------------------------------------------------------

| Mean Std. err. [95% conf. interval]

--------------+------------------------------------------------

Threshold svv | 12.15195 .2768858 11.60878 12.69512

---------------------------------------------------------------

**Supplemental Figure 1. Pooled AUC of PPV in studies with closed abdomen and chest.**

**Supplemental Figure 2. Pooled AUC of PPV in studies with closed chest and open abdomen.**


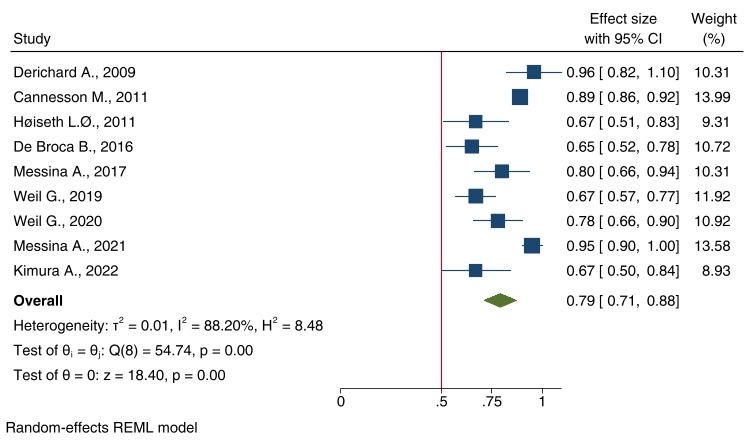


**Supplemental Figure 3. Pooled AUC of PPV in studies with closed abdomen and open chest.**

**Supplemental Figure 4. Pooled AUC of PPV in studies enrolling patients during laparoscopy**

**Supplemental Figure 5. Pooled AUC of PPV in studies enrolling patients in prone position**

 **s**

**Supplemental Figure 6. Pooled AUC of SVV in studies with closed abdomen and chest.**

**Supplemental Figure 7. Pooled AUC of SVV in studies with closed chest and open abdomen.**

**Supplemental Figure 8. Pooled AUC of SVV in studies with closed abdomen and open chest.**

**Supplemental Figure 9. Pooled AUC of SVV in studies enrolling patients during laparoscopy**

**Supplemental Figure 10. Pooled AUC of SVV in studies enrolling patients in prone position**

 **s**
